# Electroacupuncture ameliorates intestinal inflammation by activating α7nAChR-mediated JAK2/STAT3 signaling pathway in postoperative ileus

**DOI:** 10.7150/thno.52574

**Published:** 2021-02-19

**Authors:** Na-Na Yang, Jing-Wen Yang, Yang Ye, Jin Huang, Lu Wang, Yu Wang, Xin-Tong Su, Ying Lin, Fang-Ting Yu, Si-Ming Ma, Ling-Yu Qi, Lu-Lu Lin, Li-Qiong Wang, Guang-Xia Shi, Hong-Ping Li, Cun-Zhi Liu

**Affiliations:** 1 International Acupuncture and Moxibustion Innovation Institute, Beijing University of Chinese Medicine.; 2 Department of Integration of Chinese and Western Medicine, School of Basic Medical Sciences, Peking University, Beijing, China.

**Keywords:** gastrointestinal motility, macrophages, α7nAChR, JAK2/STAT3 signaling pathway, GABA_A_ receptor

## Abstract

Inflammatory cytokines produced by muscularis macrophages largely contribute to the pathological signs of postoperative ileus (POI). Electroacupuncture (EA) can suppress inflammation, mainly or partly via activation of vagal efferent. The goal of this study was to investigate the mechanisms by which EA stimulation at an hindlimb region ameliorates inflammation in POI.

**Methods:** Intestinal motility and inflammation were examined after 24 h after intestinal manipulation (IM)-induced POI in mice. Local immune response in the intestinal muscularis, expression of macrophages, α7 nicotinic acetylcholine receptor (α7nAChR), Janus kinase 2 (JAK2) and signal transducer and activator of transcription 3 (STAT3) were determined by flow cytometry, Western Blot, qPCR and immunofluorescence. The effects of α7nAChR antagonists (methyllycaconitine and α-bungarotoxin) and JAK2/STAT3 inhibitors (AG490 and WP1066) were also administered in a subset of mice prior to EA. In the parasympathetic pathways, intestinal motility and inflammation were determined after cervical vagotomy and sub-diaphragmatic vagotomy. The expression of gamma absorptiometry aminobutyric acid (GABA_A_) receptor in dorsal motor nucleus of vagal (DMV) cholinergic neurons was assessed by immunofluorescence and the response to DMV microinjection of bicuculine (antagonist of GABA_A_ receptor) or muscimol (agonist of GABA_A_ receptor) were assessed.

**Results:** EA suppressed intestinal inflammation and promoted gastrointestinal motility. Mechanistically, EA activated the α7nAChR-mediated JAK2/STAT3 signaling pathway in macrophages which reduced the production of inflammatory cytokines. Furthermore, we also demonstrated that hindlimb region stimulation drove vagal efferent output by inhibiting the expression of GABA_A_ receptor in DMV to ameliorate inflammation.

**Conclusions:** The present study revealed that EA of hindlimb regions inhibited the expression of GABA_A_ receptor in DMV neurons, whose excited vagal nerve, in turn suppressed IM-induced inflammation via activation of α7nAChR-mediated JAK2/STAT3 signaling pathway.

## Introduction

Postoperative ileus (POI) is characterized by a transient impairment of gastrointestinal motility 1, which is a major factor of morbidity after abdominal surgery and contributes to significant health care costs. The intestinal inflammation, triggered by muscularis macrophages, is the main factor responsible for the prolonged dysmotility of the gastrointestinal tract associated with POI 2. Tissue-resident macrophages are highly specialized phagocytes that carry out supportive functions during gastrointestinal development, homeostasis, and regeneration 3. In the steady state, macrophages play a crucial role in protecting the host against harmful microorganisms and continuously phagocytose and clear luminal antigens that occasionally breach the epithelial layer 4. Not surprisingly, dysregulation of tissue-resident macrophage response can also cause immune pathology. In POI, macrophages directly drive the intestinal inflammation by excessive release of pro-inflammatory cytokines, referred to as the cytokine storm 5. Exhaustion and inactivation of these macrophages have been shown to reduce intestinal inflammation 6 and may prevent POI in the muscular layer.

Vagal nerve stimulation has emerged as a potential therapeutic regimen to treat the inflammatory response 7, 8. Electric stimulation of cervical vagal efferent has been shown to suppress systemic inflammation by activation of α7 nicotinic acetylcholine receptor (α7nAChR) found on tissue-resident macrophages and other immune cells 9, 10. The unique anti-inflammatory function of the vagal nerve opens the possibility of using vagal nerve stimulation as a means of reducing pathological intestinal inflammation in POI 11.Recently, electroacupuncture (EA) has emerged as a non-invasive method of stimulating the vagal nerve, and therefore as a potential means of reducing pathological intestinal inflammation 12.

A core principal of Traditional Chinese Medicine (TCM) is that specific somatic tissues (acupoints) are related to visceral and systemic conditions, and stimulating these acupoints can modulate organ physiology through meridian channels 13. Although modern anatomical studies failed to support the physical presence of such “channels” 14, somatosensory autonomic pathways could serve this role 15. Inspired by the discovery of cholinergic anti-inflammatory pathways, several studies showed that limb area acupoint stimulation can suppress the inflammatory response, mainly or partly via activation of vagal efferent 16. We previously found that EA at ST36 also can alleviate intestinal inflammation and promote gastrointestinal motility in POI 17.

Despite this progress, many challenges and knowledge gaps remain in studying somatosensory parasympathetic pathways. First, it remains unclear that as a body distal stimulation, how does EA drive the vagal nerve in POI. Second, it remains to be determined how the inflammatory response is suppressed by EA-activated vagal nerve. As such, the goal of this study was to investigate how does stimulation at the hindlimb region drives vagal nerve to ameliorate inflammation.

Here we reported that EA improves gastrointestinal motility and reduces the intestinal inflammatory cytokines via activation of α7nAChR-mediated Janus kinase 2/signal transducer and activator of transcription 3 (JAK2/STAT3) signaling pathway in POI. Moreover, EA at the hindlimb regions inhibits the expression of gamma absorptiometry aminobutyric acid (GABA_A_) receptor in dorsal motor nucleus of vagal (DMV) neurons, whose excited vagal nerve, in turn suppresses intestinal manipulation (IM)-induced inflammation.

## Methods

### Mice

C57BL/6 mice of 20-25g weight (Vital River Laboratory Animal Technology Co., Ltd, Beijing, China) were maintained under specific pathogen-free conditions on a 12 h light/dark cycle and fed rodent chow and tap water ad libitum. All experiments were performed following International Guidelines of using Animals in Scientific Procedures. The principles of laboratory animal care were followed; the Institutional Animal Care and Use Committee (IACUC) approved animal activities under the approval number BUCM-4-2018101901-4009.

### Surgery

POI was induced in WT C57BL/6 mice by IM as described previously 18, 19. Briefly, mice were anesthetized with pentobarbital sodium solution (1%, 50mg/kg, i.p), followed by a midline abdominal incision was made into the peritoneal cavity. The small bowel was carefully exteriorized and then manipulated along its entire length by using two moist cotton applicators. Finally, the bowel was repositioned, and the abdomen was closed by a two-layer of suture. Mice in the sham operation group exclusively underwent laparotomy without IM.

### EA Intervention

During the EA stimulation, mice were under anesthesia and were loosely immobilized by an assistant's hands. EA intervention was applied for 20 min under anesthesia. Two stainless steel acupuncture needles (0.16 × 7 mm) were inserted at a depth of 3 mm into the bilateral ST36 acupoint. The ST36 acupoint is located 2 mm lateral to the anterior tubercle of the tibia and 3 mm below the knee joint.

The electrical stimuli were administered using a HANS-200A Acupuncture Point Nerve Stimulator (Nanjing, China) at an intensity of 1 mA, 10 Hz, plus width 0.4 ms, 20 min, which, in our experience, is the threshold to detect a muscle twitch. In the non-acupoint group, needles were inserted to a depth of 3 mm into the tail at its mid-point, a location not corresponding to any traditional acupuncture point but were given the same electrical stimulation.

### Selective neurectomies

Selective neurectomies were performed before EA intervention. *Sciatic neurectomy*: The mice were anesthetized and the hindlimb was shaved and sterilized. The skin incision was made posterior to the femoral trochanteric region. The sciatic nerve was exposed and a 1-cm-long segment was resected before the EA treatment, as described in Ishimaru et al 20. In the sham group mice were anesthetized and incised in the same manner as the sciatic neurectomy group but without sciatic neurectomy. *Cervical vagotomy*: It was performed as described by Vida et al 12. Animals were subjected to an anterior incision of the neck to access the sternocleidomastoid muscle. The sternocleidomastoid muscle was dissected to visualize the carotid artery and the vagal nerve. The vagal branches of the left neck were stabilized with nylon thread and cut. In the sham group mice were anesthetized and incised in the same manner as the cervical vagotomy group but without cervical vagotomy. *Sub-diaphragmatic vagotomy:* It was performed as described by Huston et al 21. Animals underwent an abdominal incision covering the epigastrium and mesogastrium. The esophagus was exposed at juncture to the stomach under a surgical microscope. Subsequently, the ventral and dorsal branches were exposed, and a 5-mm length of each branch was resected beneath the diaphragm in the vagotomy group. The sham group mice were anesthetized and incised in the same manner as the sub-diaphragmatic vagotomy group, but without sub-diaphragmatic vagotomy.

### Microinjection

Mice were anesthetized and placed in a stereotaxic apparatus. Following skin incision and cleaning the skull, bilateral micropipettes were placed. The stereotaxic coordinates for DMV are AP: - 7.2 mm from bregma, L: ± 0.5 mm from the sagittal suture, and V: 4.5 mm from the skull surface. The micropipettes were connected to a 0.5 ul Hamilton microsyringe. The guide cannulas were secured using dental acrylic. All animals were allowed 1 week to recover from surgery and the effects of anesthetic drugs. As a routine, bicuculine, an inhibitor of GABA_A_ receptor, 2 µg/µl, or muscimol, an agonist of GABA_A_ receptor, 1µg/µl was delivered to each side of the DMV over 1-2 min to allow for complete diffusion of the test agents before EA treatment 22.

### Evaluation of Intestinal Motility

Intestinal motility was assessed 24 h postoperatively *in vivo* by evaluating the intestinal location of fluorescein labeled dextran (FITC-dextran), as previously described 19. FITC-dextran (70.000 kDa, ThermoFisher) dissolved in 0.9% saline (50 mg/ml) were administered via oral gavage 22.5 h after surgery. After 90 min, the mice were sacrificed and the entire gastrointestinal tract was divided into 15 segments (1: stomach, 2-11: ten equal parts from the small bowel, 12: cecum and 13-15: three equal parts of the colon). The contents of the gastrointestinal tract were collected in tubes containing Krebs-Henseleit buffer solution. Finally, the fluorescence intensity in each intestinal segment was measured using a microplate reader (Thermo Fisher Scientific, USA) with an excitation wavelength of 494 nm and emission wavelength of 521 nm. Gastrointestinal transit was calculated as the geometric center (GC) of FITC dextran distribution by the following formula: GC = ∑ (% of total fluorescent signal per segment × segment number) /100.

### ELISA

The tumor necrosis factor α (TNF-α) and Interleukin 6 (IL-6) levels in serum were measured by enzyme-linked immunosorbent assay (ELISA) assay. Blood was collected from each mouse at the time of sacrifice and the blood sample was centrifuged to separate the serum. The serum samples were stored at -80 °C until assays were performed. Cytokines were analyzed by ELISA according to the manufacturers' protocol.

### qPCR

Total RNA from tissue was isolated with Trizol (Invitrogen) and then was reverse transcribed using ReverTraAce qPCR RT kit (Toyobo). qPCR was performed on the CFX96 Touch Real-Time PCR Detection System (Bio-Red) using SYBR Green reagent (Roche). Primer sequences are listed in Table S1.

### Western Blot

Standard western blot techniques were used on the isolated jejunal muscularis externa. The membranes were blocked with 5% skimmed milk and incubated with primary antibodies (α7nAChR, 1:500; JAK2, 1:500; P-JAK2, 1:500; STAT3, 1:500; P-STAT3, 1:1000; β-actin, 1:2000) from Cell Signaling Technology. Anti-rat IgG (1:1500) and anti-rabbit IgG (1:1000) were used for secondary antibodies from Cell Signaling Technology.

### Immunofluorescence

Jejuna and brain sections were blocked with goat serum and incubated with rat anti-mouse primary antibodies to F4/80 (1:200), cholinergic (ChAT) neurons (1:1000)and rabbit anti-mouse primary antibodies α7nAChR (1:200), P-JAK2 (1:200), P-STAT3 (1:100) GABA receptor (1:1000) at 4 °C overnight. Subsequently, sections were incubated for 1 h at room temperature in the dark with Alexa Fluor 488-labeled secondary antibody (1:1000) or Alexa Fluor 647-labeled secondary antibody (1:1000).

### Flow Cytometry

Single-cell was prepared from the small intestine as previously described 23. In brief, the intestine was longitudinally opened, cut into 3-inch segments, and incubated in RPMI with 5% dithiothreitol (DTT), 0.5 M EDTA and fetal bovine serum (FBS) at 37 °C to remove epithelial cells. Tissue was digested with dispase and collagenase II in a complete RPMI medium containing FBS for 30 min at 37 °C. The tissue was then mechanically dissociated by gentle trituration and filtered through a 40 µm nylon mesh cell strainer.

10^5^-10^6^ cells were stained as described previously using the antibodies listed in Table S2 followed by blocking with mouse anti-CD16/CD32 (BD Biosciences). After surface staining, cells were permeabilized using Fixation/Permeabilization solution (BD Biosciences). After a further stain with AF-647 anti-p-JAK2 for 30 min, cells were washed before sample acquisition. It is distinguished by the expression of CD11c, CD11b, CD64, F4/80, Ly6G and Ly-6C for myeloid cells and CD3, CD4 and CD8 for T cells population. All stained samples were acquired using a LSRFortessa (BD Biosciences) and analyzed using FlowJo software.

### Statistical Analysis

Data were reported as means ± SEM. Statistical analysis was performed using SPSS 23.0 software. To compare multiple groups, one-way analysis of variance followed by the Bonferroni post-hoc test was performed. A *P* value of < 0.05 was considered statistically significant.

## Results

### EA ameliorated the delay of gastrointestinal transit and inhibited the production of cytokine

**Figure 1:**
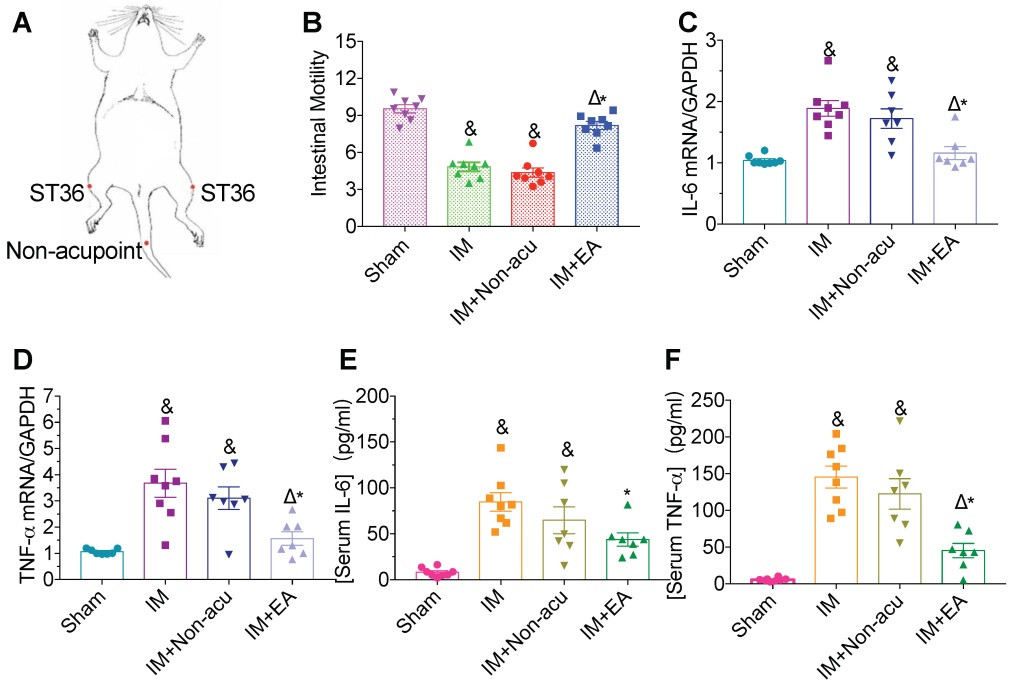
** EA attenuates IM-induced motility dysfunction and inflammatory in serum and intestinal muscularis.** (A) Schematics showing the hindlimb ST36 acupoint and non-acupoint. (B) The mean gastrointestinal transit was calculated 24h after surgery. The expression of IL-6 (C) and TNF-α (D) in intestinal muscularis was analyzed by qPCR. The expression of serum IL-6 (E) and TNF-α (F) was analyzed by ELISA. ^&^*p* < 0.05 versus Sham group, ^*^p < 0.05 versus IM group, ^Δ^*p* < 0.05 versus IM + Non-acu group.

We adopted IM-induced POI as a model to study the effect of EA in gastrointestinal motility and inflammation at the hindlimb ST36 acupoint (Figure 1A). In the sham group, FITC-dextran moved rapidly, with the peak fluorescence signal in the ninth segment of the small bowel (GC = 9.27 ± 0.35) and the highest fluorescence intensity was in the more proximal segments in the IM group (GC = 5.14 ± 0.49, Figure 1B). Likewise, IM-induced POI significantly increased IL-6 and TNF-α, both in serum and intestinal tissues compared with the sham group (Figure 1C-F). EA significantly improved gastrointestinal transit (GC = 7.93 ± 0.43) compared with the IM group (Figure 1B) and decreased surgery-induced pro-inflammatory cytokine serum levels and their expression in the intestinal muscularis (Figure 1C-F), but not by non-acupoint EA.

### α7nAChR expression in the macrophages was essential for the anti-inflammatory effect of EA in POI

**Figure 2:**
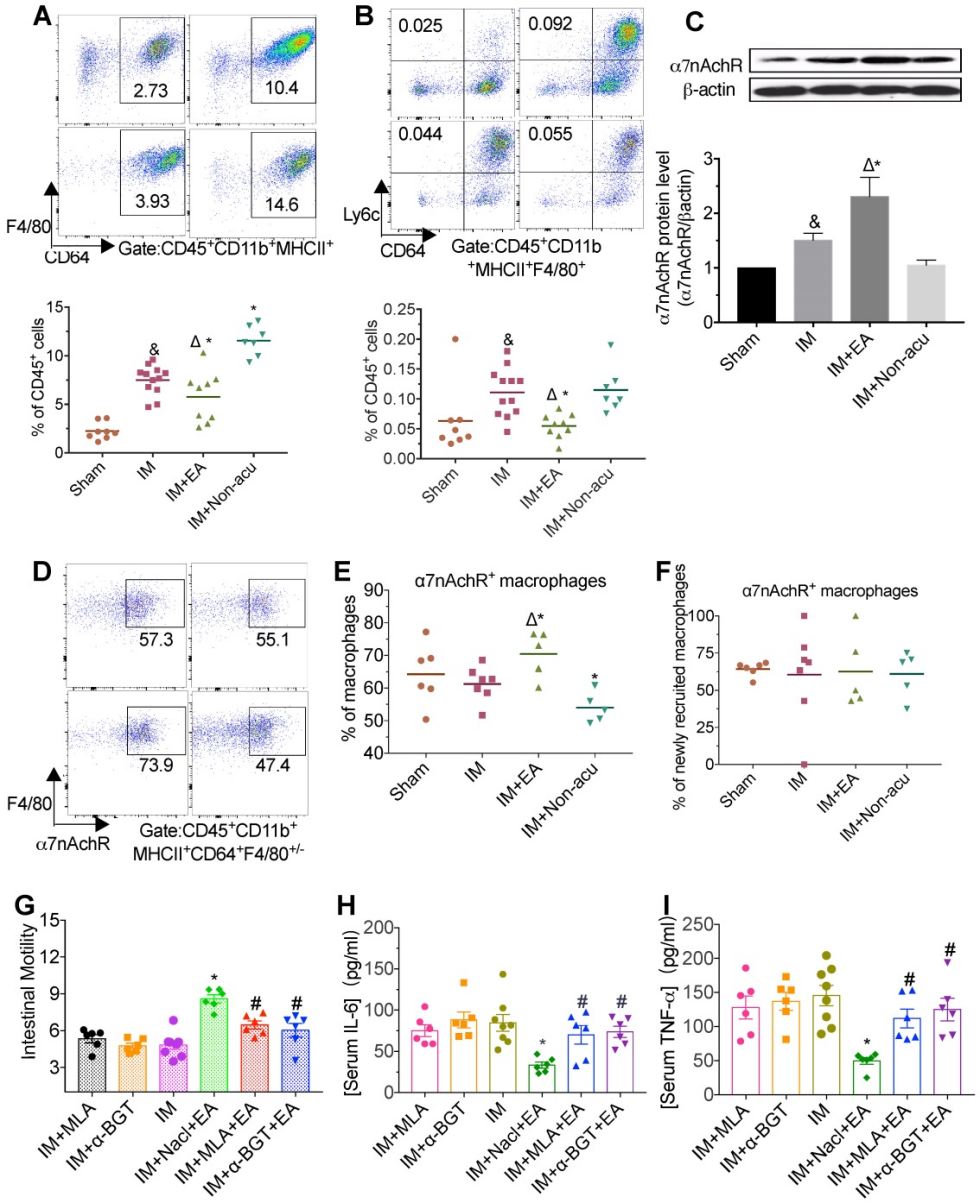
**The anti-inflammatory effect of EA was medicated by α7nAChR.** The frequencies of CD45^+^CD11b^+^MHCII^+^CD64^+^F4/80^+/-^ macrophages (A) and newly recruited macrophages (B) among CD45^+^ cells by flow cytometry. The expression of α7nAChR in intestinal muscularis was evaluated by Western blot (C). The frequencies α7nAChR^+^ macrophages (D.E) or newly recruited macrophages (F). The mean gastrointestinal transit (G) and the level of serum TNF-α (H) and IL-6 (I) was analyzed by ELISA. ^&^*p* < 0.05 versus Sham group, ^*^p < 0.05 versus IM group, ^Δ^*p* < 0.05 versus IM + Non-acu group, ^#^*p* < 0.05 versus IM + Nacl + EA group.

The intestinal muscularis is densely populated by T cells and myeloid cells including macrophages, dendritic cells (DCs) and monocytes, all of which contribute to the maintenance of tissue homeostasis and integrity but are probably especially pertinent in intestinal inflammation 24. To explored how EA modulated inflammation, we focused on these immune cells, especially in macrophages. Standard markers were used to discriminate macrophages (CD64^+^F4/80^+/-^), newly recruited macrophages (CD64^-^F4/80^+^Ly6C^+^) and CD11b^+^ DCs (CD64^-^ F4/80^-^CD11c^+^) from CD11b^+^MHCII^+^ single live leukocytes. The typical marker of CD11b^-^ MHCII^+^CD11c^+^ was for CD11b^-^ DCs, meanwhile, the CD11b^+^MHCII^-^ cells polymorphonuclear neutrophils (PMN) and monocytes are then identified by Ly6G and Ly6C. IM-induced POI resulted in an increased numbers of myeloid cells (CD45^+^CD11b^+^) in the intestinal muscularis compared to sham-IM (Figure S1A). This increasing in myeloid cells seen in the IM group was reduced by EA at hindlimb ST36 acupoint, especially Ly6c^+^ myeloid cells (Figure S1A). Meanwhile, the percentage of macrophages, including newly recruited macrophages, was significantly higher in CD45^+^cells of the IM group (Figure 2A-B). The accumulation of macrophages in muscularis was markedly suppressed by EA, but not non-acupoint (Figure 2A-B and Figure S1B).

The percentage of DCs including CD11b^+^ or CD11b^-^, CD4^+^T cells and CD8^+^ T cells among CD45^+^ leukocytes were not significantly different in the four groups (Figure S1C-E). Besides, compared with the IM group, EA led to a reduction of the monocytes and PMN percentage (Figure S1F and Figure S2A). These data suggested that the accumulation and activation of intestinal macrophages were the key target in EA treatment.

Macrophages express the highest level of α7nAChR 25 and several preclinical studies of systemic inflammation have convincingly demonstrated that the vagal nerve anti-inflammation pathway requires the presence of α7nAChR 12. To determine whether α7nAChR is involved in the anti-inflammatory effects of EA, we next measured the concentration of α7nAChR after EA. Twenty-four hours after IM, expression of α7nAChR was markedly increased by EA to hindlimb acupoint, but not by EA to tail non-acupoint (Figure 2C and Figure S2B). To further characterize the relationship between macrophages and α7nAChR, we assessed the expression of α7nAChR in macrophages and non-macrophage cells by flow cytometry. Macrophages in intestine expressed lower amounts of α7nAChR, which was reversed by EA, but not by non-acupoint (Figure 2D-F). Meanwhile, the expression of this receptor in PMN and monocytes were also increased by EA (Figure S2D-E). In contrast, no significant difference in α7nAChRexpression was seen in T cells (Figure S2F-G). With consideration to the co-location of α7nAChR and macrophages (Figure S2C), these data confirmed that functional α7nAChR was mainly expressed on the surface of macrophages which were modulated by EA.

EA can suppress pro-inflammatory cytokines production in intestinal macrophages via activation of α7nAChR. We next asked whether blockage of α7nAChR by the antagonist methyllycaconitine (MLA) and α-bungarotoxin (α-BGT) could affect EA-evoked anti-inflammatory effects. We found that *in vivo* injection of MLA or α-BGT recaptured the phenotypes seen in POI mice, including (1) dampening the improvement of gastrointestinal transit by EA (Figure 2G), (2) a loss of effects by ST36 EA in reducing overall TNF-α and IL-6 production (Figure 2H-I and Figure S2H-I). These studies suggest that EA at the hindlimb regions activated α7nAChR, whose inhibited intestinal macrophages infiltration, in turn suppressed IM-induced inflammation.

### α7nAChR-mediated JAK2/STAT3 signaling pathway in macrophages was activated by EA stimulation

**Figure 3:**
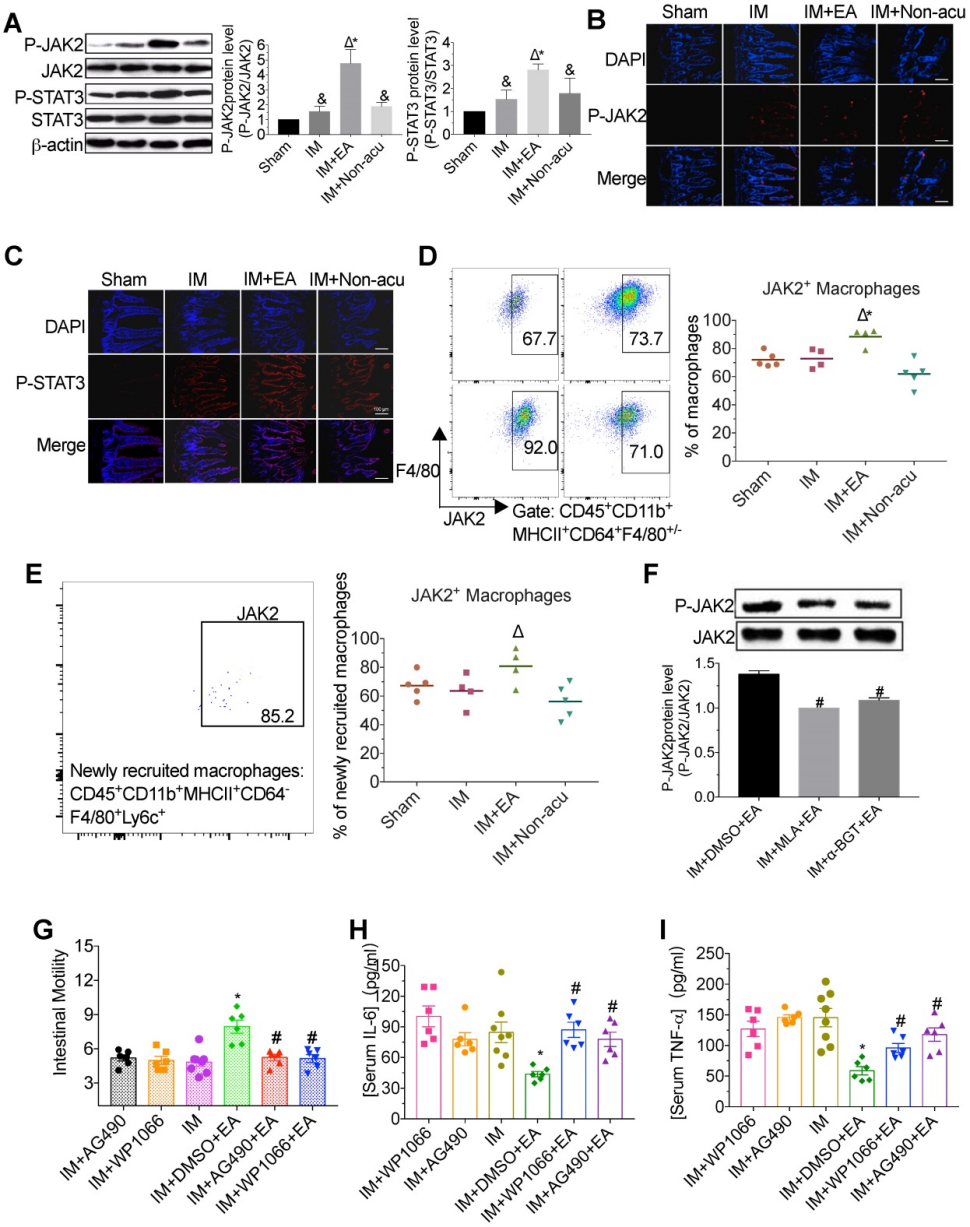
**EA stimulation activated the α7nAChR-mediated JAK2/STAT3 pathway in macrophages.** The level of JAK2 and STAT3 in intestinal muscularis was evaluated by Western blot (A) or immunofluorescence staining (B, C). × 100 magnification. The frequencies JAK2^+^ macrophages or newly recruited macrophages (D, E). The expression of JAK2 was evaluated by Western blot with α7nAChR antagonist MLA and α-BGT (F). The mean gastrointestinal transit was evaluated in each group (G). The expression of serum IL-6 (H) and TNF-α (I) was analyzed by ELISA. ^&^*p* < 0.05 versus Sham group, ^*^p < 0.05 versus IM group, ^Δ^*p* < 0.05 versus IM + Non-acu group, ^#^*p* < 0.05 versus IM + DMSO + EA group.

To elucidate the underlying cellular mechanism of EA stimulation, we analyzed whether EA at ST36 augmented JAK2/STAT3 activation, which is known to be a crucial role in the cholinergic anti-inflammatory pathway 26. We found that IM resulted in the increasing appearance of phosphorylated JAK2 and STAT3, and interestingly, EA stimulation significantly enhanced this process, whereas EA at non-acupoint did not produce a significant improvement (Figure 3A-C). To illustrate that EA showed a selective activation JAK2/STAT3 signaling in intestinal macrophages, the level of JAK2 was determined in macrophages and non-macrophage cells by flow cytometry. Compared with model mice, the expression of JAK2 in macrophages including newly recruited macrophages was higher after EA, which was similar in PMN and monocytes (Figure 3D-E and Figure S3A-B). To further determine whether the activation of the JAK2/STAT3 pathway was medicated by α7nAChR, we determined expression of JAK2 with α7nAChR antagonist MLA and α-BGT. We found that α7nAChR antagonists prevented the JAK2 phosphorylation induced by EA (Figure 3F).

To evaluate whether JAK2 and STAT3 phosphorylation are crucial to the anti-inflammatory effect of EA, we abrogated JAK2 or STAT3 phosphorylation by intraperitoneal injection AG490 (a well-known inhibitor for JAK2) or WP1066 (a well-known inhibitor for STAT3). Compared with the IM group, EA increased the gastrointestinal motility; however, such improvement was abolished by AG490 or WP1066 (Figure 3G). Consistently, although EA led to a marked reduction of IL-6 and TNF-α levels in serum and intestinal muscularis in the DMSO group compared with IM group, no such inflammatory amelioration was observed in AG490 or WP1066 group (Figure 3H-I and Figure S3C-D). These findings indicated that EA increased the expression of α7nAChR on macrophages to control inflammation via activation of JAK2/STAT3 signaling pathway.

### The protective function of EA was abolished by vagotomy in POI

**Figure 4:**
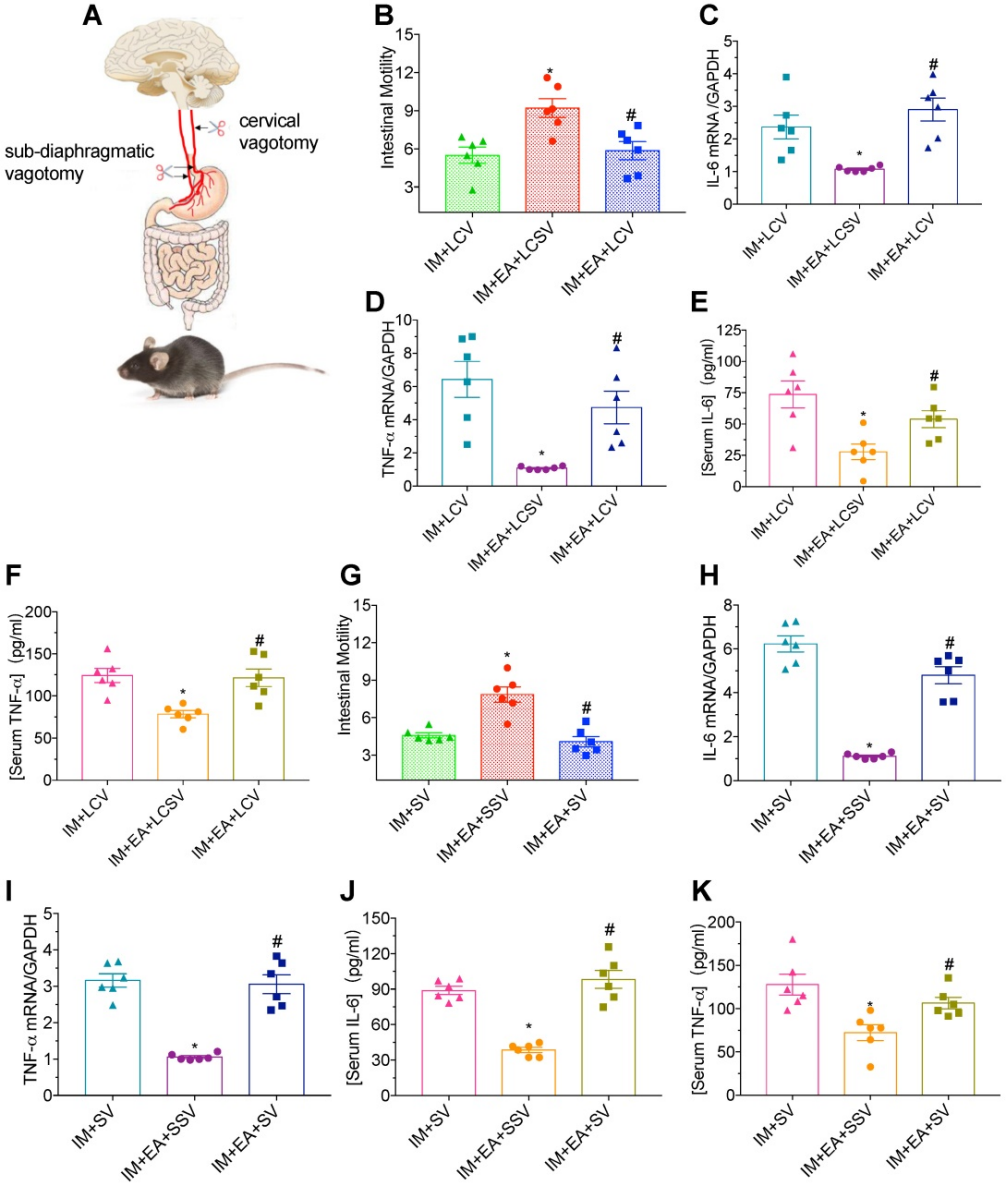
** The protective function of EA was nearly abolished by vagotomy in POI.** (A) Schematic showing the location of vagotomy. (B and G) The mean gastrointestinal transit was evaluated after LCV or SV. The expression of intestinal muscularis IL-6 (C, H) and TNF-α (D, I) was analyzed by qPCR. The expression of serum IL-6 (E, J) and TNF-α (F, K) was analyzed by ELISA.^ *^p < 0.05 versus IM + LCV or IM + SV group,^ #^*p* < 0.05 versus IM + EA + LCSV or IM + EA + SSV group. LCV, left cervical vagotomy; LCSV, left cervical sham vagotomy; SV, sub-diaphragmatic vagotomy; SSV, sub-diaphragmatic sham vagotomy.

Vagal nerve-mediated cholinergic signaling controls immune functions and the inflammatory response 27. To determine whether the vagal nerve is essential for the immunomodulatory function of EA in IM-induced POI, we performed a left cervical vagotomy or sub-diaphragmatic vagotomy after IM. Neither gastrointestinal transit (Figure 4B and G) nor a reduction of TNF-α and IL-6 (Figure 4C-F and H-K) evoked by EA at ST36 was affected in mice with a left cervical vagotomy or sub-diaphragmatic vagotomy compared with sham surgery. Taken together, these findings indicated that vagal nerve was required for EA at ST36 to modulate the gastrointestinal motility and α7nAChR mediated inflammatory response in POI.

We next explored how EA modulated systemic information, by focusing on the somatosensory vagal pathways. The ST36 acupoint is in the proximity of the common peroneal and tibial branches of the sciatic nerve. We found that sectioning of the sciatic nerve abolished the increased motility and anti-inflammatory effects of EA in POI (Figure S4A-E). Meanwhile, EA at the hindlimb ST36 acupoint was able to induce c-Fos expression in the lumbar spinal cord (Figure S4F) indicating the activation of the somatosensory system.

### Vagal nerve excitation via EA was regulated by GABA_A_ receptors in DMV cholinergic neurons

**Figure 5:**
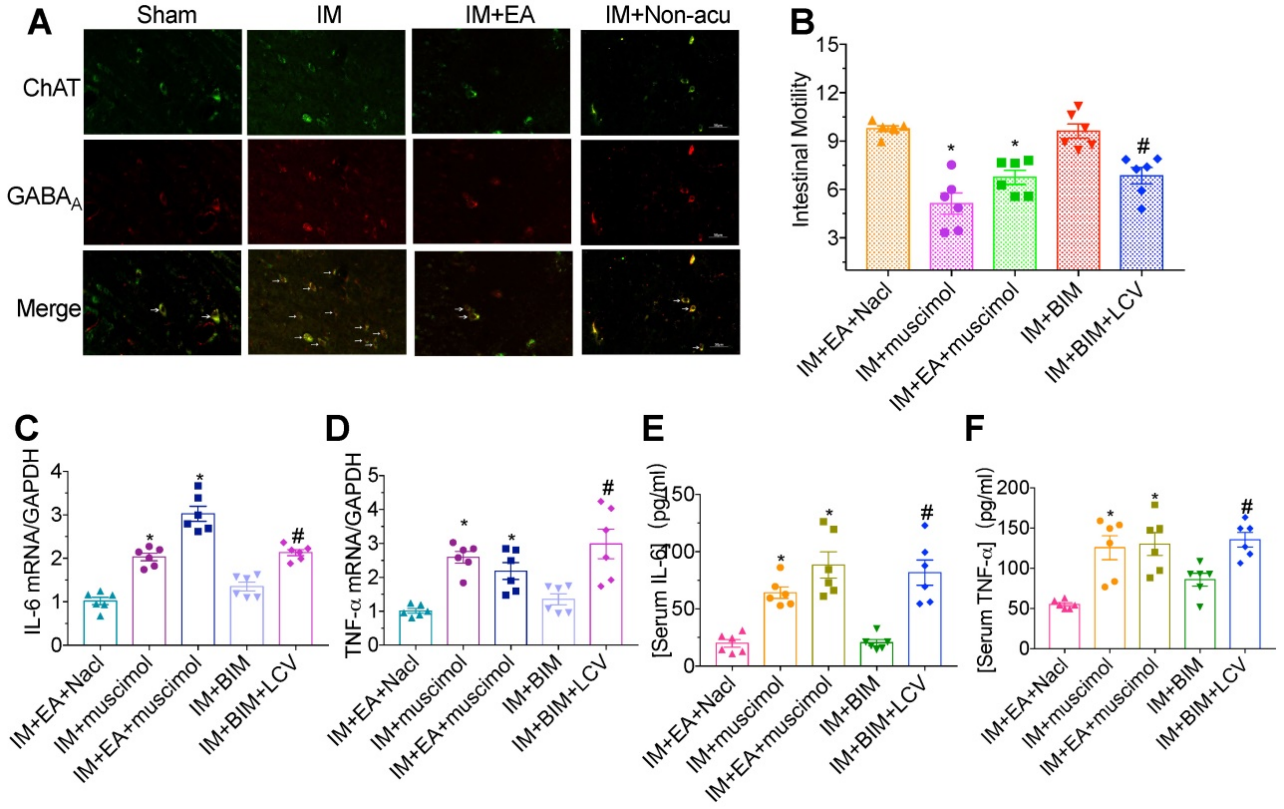
**Vagal nerve activity was mediated by GABA_A_ receptors in ChAT.** The level of GABA_A_ was evaluated by immunofluorescence staining(A). × 400 magnification. (B) The gastrointestinal transit was measured in each brain microinjection group. The expression of IL-6 (C) and TNF-α (D) in intestinal muscularis was analyzed by qPCR. The expression of serum IL-6 (E) and TNF-α (F) was analyzed by ELISA. ^*^p < 0.05 versus IM + EA + Nacl group,^ #^*p* < 0.05 versus IM + BIM group. BIM, bicuculline.

Activation of subunit-specific γ-aminobutyric acid A (GABA_A_) receptor in the DMV inhibits vagal output activity to the gastrointestinal system, which reduces gastrointestinal motility. We explored whether the vagal nerve activity originating from EA stimulation was mediated by GABA_A_ receptor. The majority of cholinergic neurons in the DMV from the IM group showed a strong colocalization with GABA_A_ receptor and exhibited high GABA_A_ receptor fluorescence intensity. EA at ST36 was sufficient to inhibit the expression of GABA_A_ receptor in hindbrain ChAT^+^ vagal efferent neurons located in DMV (Figure 5A), indicating that the excitation of vagal nerve resulted from EA was mediated by GABA_A_ receptor in DMV cholinergic neurons.

To further address the role of GABA_A_ receptor on the brainstem control of gastrointestinal transit, bicuculline and muscimol were microinjected into the DMV. Gastrointestinal motility decreased and severe inflammatory response were again observed after EA with muscimol microinjection compared with EA plus NaCl group (Figure 5B). However, microinjections of bicuculline could mimic the effects of EA on gastrointestinal motility and inflammatory response, which was nearly abolished by left cervical vagotomy. Taken together, these findings indicated that EA at hindlimb regions inhibited the expression of GABA_A_ receptor in DMV ChAT^+^ neurons to modulate the gastrointestinal motility and inflammatory response in POI (Figure 5C-F).

## Discussion

Dysregulation of the intestinal immune system plays a major role in the pathophysiology of POI. Muscularis macrophages, although quiescent under normal physiological conditions, act as pro-inflammatory cells during the first 24 h after IM, producing the pro-inflammatory cytokines and increasing the infiltration of immune cells from the circulation. Monocytes which could replenish the macrophage populations in the intestine and neutrophils enter the intestinal muscularis, further recruiting more pro-inflammatory macrophages thereby further contributing to POI by releasing factors such as IL-6 and TNF-α 28. Here we shown that only 2% of myeloid cells expressed Ly6c in the sham group, in line with the finding that infiltrating immune cells are rarely encountered in the quiescent intestine. Myeloid cells, particularly monocytes, were more abundant after IM, indicating that immune dysregulation plays a central role in POI. Importantly, EA activated the α7nAChR-mediated JAK2/STAT3 signaling pathway in macrophages, PMN and monocytes. EA also decreased the percentage of PMN, monocytes and macrophages among CD45^+^ leukocytes. In addition, EA also suppressed newly recruited macrophages in the muscularis. Therefore, it is a reasonable assumption that EA at ST36 acupoint appears to reduce IM-induced POI inflammation by suppressing the activation of resident macrophages, thereby decreasing the recruitment of circulating monocytes and neutrophils, and in turn, suppress the accumulation of newly recruited macrophages and decrease the level of inflammatory cytokines. Collectively, these results indicated that macrophages are a major constituent of the EA-driven anti-inflammation pathway in POI.

The α7nAChR, which is activated by acetylcholine released from the cholinergic nerve endings, is the crucial target for attenuation of pro-inflammatory cytokines released from macrophages 29. Bone marrow chimera studies proved that the α7nAChRs were localized to immune cells rather than ganglia in the enteric nervous system. Meanwhile, macrophages were found to express much higher levels of α7nAChR mRNA than other immunes cells, which was similar to our results 25. In terms of the improvement of POI, Hong et al 30 showed that the activation of α7nAChR on macrophages could inhibit intestinal wall inflammation. Similarly, our results indicated that the deactivation of macrophages, which was mediated by α7nAChR upregulation, contributed to the anti-inflammatory effect of EA as demonstrated by reversal of this anti-inflammatory effect when EA at ST36 was performed in the presence of the α7nAChR-specific antagonist, MLA or α-BGT. Thus, EA acted directly through enteric cholinergic neurons to regulate immune function in the gastrointestinal tract.

Although it is not entirely understood, α7nAChR stimulation leads to the recruitment of JAK2 to α7nAChR and then JAK2 aggregates and phosphorylates STAT3 (p-STAT3), and finally p-STAT3 forms a dimer and transfers to the nucleus 26, which negatively regulates inflammatory cytokine. Our study indicated that increasing of JAK2/STAT3 tyrosine phosphorylation by EA restrained the inflammatory responses and increased motility in POI model. LysMcre/Stat3^flox/-^ mice which were designed for cell-specific STAT3 disruption in macrophages and neutrophils had overwhelming inflammatory responses to bacterial endotoxin, which made them very susceptible to endotoxemia and sepsis 31. Similar to STAT3 knockout mice, our results with JAK2 inhibitor AG490 or STAT3 inhibitor WP1066 confirmed that inhibition of JAK2/STAT3 protein expression almost prevented the effects of EA and enhanced inflammatory. Furthermore, the activation of JAK2/STAT3 pathway by EA was reversed by MLA or α-BGT. The greater expression of this signaling pathway in the IM group might be associated with the physiological anti-inflammatory pathway which triggered compensatory activation during IM and the underlying cellular mechanism behind the EA is probably multifactorial, involving NF-κB and p38-MAPK pathways 32, 33. These data lend support to our hypothesis that EA attenuated POI, at least in part, via the activation of α7nAChR-mediated JAK2/STAT3 signaling pathway in macrophages.

The vagal nerve acts as a bridge between the neural and immune systems and can activate the α7nAChR on resident macrophages. Notably, vagal activity has been previously implicated as a possible mediator for acupuncture therapy 34. In our study both cervical and sub-diaphragmatic vagotomy abolished the anti-inflammatory potential of EA; these results indicate that the vagal activity caused by EA and vagal nerve stimulation may share some common neural code in acting on a target organ. Similarly, a recent study showed that low-intensity stimulation of the hindlimb ST36 acupoint produced anti-inflammatory effects that depend on vagal efferent associated with DMV neurons 15. However, another study 35 showed that EA with 2 Hz treatment partly promoted the recovery time of POI by activating the vagal nerve but not regulating local inflammation. We found that EA at 10Hz could attenuate inflammation and promote gastrointestinal function in POI 36. It worth noting that the effect of EA in vagal nerve excitation 35 and anti-inflammation 36 were influenced by stimulation frequency. The difference in stimulation frequency may account for the different results of EA on local inflammation, although these studies demonstrated the similar role of EA on vagal activity in POI.

EA was found to modulate gastrointestinal motility in an intensity-dependent manner, via a mechanism of sympathetic nerves 37. EA briefly increased gastrointestinal motility via adrenergic reflexes involving a spinal loop, which were afferent splanchnic nerves synapse in the spinal cord activating efferent traveling to the gut. However, the effects of EA on increasing gastrointestinal motility were almost reversed by cervical or sub-diaphragmatic vagotomy. Based on our results, the vagal nerve appears to play a prominent role in increasing gastrointestinal motility.

Anatomically, there are spinal ascending projections to the nucleus tractus solitarius (NTS) in the medulla oblongata, and NTS neurons in turn send synaptic outputs to the DMV, where vagal efferent neurons, marked by the expression of the ChAT, are located 38. There is considerable evidence that GABAergic neurons are responsible for a significant part of this communication which controls the gastric tone and motility 39. Studies from several laboratories have shown that EA significantly activated the NTS neurons after IM 16. The activity of vagal motoneurons innervating the upper GI tract is inhibited by GABAergic synaptic inputs from DMV 40. The present study suggested that the GABA_A_ receptor in DMV cholinergic neurons, and vagally-dependent gastrointestinal functions, from POI model mice, were under a greater degree of tonic synaptic inhibition which could be reversed by EA. Removal of GABAergic inputs by application of GABA receptor antagonists has been shown to increase firing rates of DMV neurons and to increase gastrointestinal tone and motility 41. In our results, muscimol microinjection in DMV reversed EA function in POI and the application of low concentrations of GABA_A_ receptor antagonist bicuculline could mimic the function of EA in increasing the motility and decreasing inflammation in muscularis; however, the effect of bicuculline could be prevented by cervical vagotomy. These findings indicate that EA might activate NTS neurons to inhibit DMV cholinergic neurons expressing GABA_A_ receptor, and consequently, vagal modulation of inflammatory reactions by EA may be augmented by a disinhibition of synaptic transmission in DMV neurocircuitry.

## Conclusion

To our knowledge, this is the first study to show a complete EA anti-inflammatory signaling pathway in POI. The present study revealed that EA at the hindlimb regions inhibited the expression of the GABA_A_ receptor in DMV ChAT neurons, whose excited vagal nerve, in turn suppresses IM-induced inflammation via activation of α7nAChR-mediated JAK2/STAT3 signaling pathway in macrophages.

## Supplementary Material

Supplementary figures and tables.

## Abbreviations

α7nAChR: α7 nicotinic acetylcholine receptor
α-BGT: α-bungarotoxin
DC: dendritic cells
DMSO: dimethyl sulfoxide
DMV: dorsal motor nucleus of vagal
EA: electroacupuncture
GABA_A_: gamma absorptiometry aminobutyric acid
IL-6: interleukin 6
IM: intestinal manipulation
JAK2: janus kinase 2
LCSV: left cervical sham vagotomy
LCV: left cervical vagotomy
MLA: methyllycaconitine
NTS: nucleus tractus solitaries
PMN: polymorphonuclear
POI: postoperative ileus
SCT: sciatic neurectomy
SCST: sciatic sham neurectomy
SV: sub-diaphragmatic vagotomy
SSV: sub-diaphragmatic sham vagotomy
STAT3: signal transducer and activator of transcription 3
TNF-α: tumor necrosis factor α
