# Supplementary material for: Electroacupuncture ameliorates intestinal inflammation by activating α7nAChR-mediated JAK2/STAT3 signaling pathway in postoperative ileus
Source: Theranostics. 2021 Feb 19;11(9):4078–89. doi: 10.7150/thno.52574 (PMC7977469; doi:10.7150/thno.52574)
Supplement: Supplementary file 1 — Supplementary figures and tables. [file thnov11p4078s1.pdf]

Table S1 Primers for qPCR analysis

Gene	Direction	Primer (5’-3’)
β-actin	F (forward)	CCTCTATGCCAACACAGTGC
211bp	R (reverse)	GTACTCCTGCTTGCTGATCC
TNF-α	F	GAGTCCGGGCAGGTCTACTTT
235bp	R	CAGGTCACTGTCCCAGCATCT
IL-6	F	TCCAGAAACCGCTATGAAGTTC
72bp	R	CACCAGCATCAGTCCCAAGA

Table S2. List of flow cytometry antibody details

Antibody	Fluorochrome	Clone	Host species	Source
CD45	BUV395	30-F11	Rat	BD Biosciences
CD3	BV510	145-2C11	Hamster	BD Biosciences
CD11c	BB700	HL3	Hamster	BD Biosciences
CD4	Pe-Cy7	RM4-5	Rat	BD Biosciences
CD8	APC-Cy7	53-6.7	Rat	BD Biosciences
CD11b	BV786	M1/70	Rat	BD Biosciences
Ly6G	BV711	1A8	Rat	BD Biosciences
Ly6C	BV605	AL-21	Rat	BD Biosciences
F4/80	BUV737	T45-2342	Rat	BD Biosciences
CD64	PE	X54-5/7.1	Mouse	BD Biosciences
MHC II	I-A/I-E BB515	2G9	Rat	BD Biosciences
α7nAchR	AF-647	/	Rabbit	Abcam
P-JAK	AF-647	/	Rabbit	Abcam

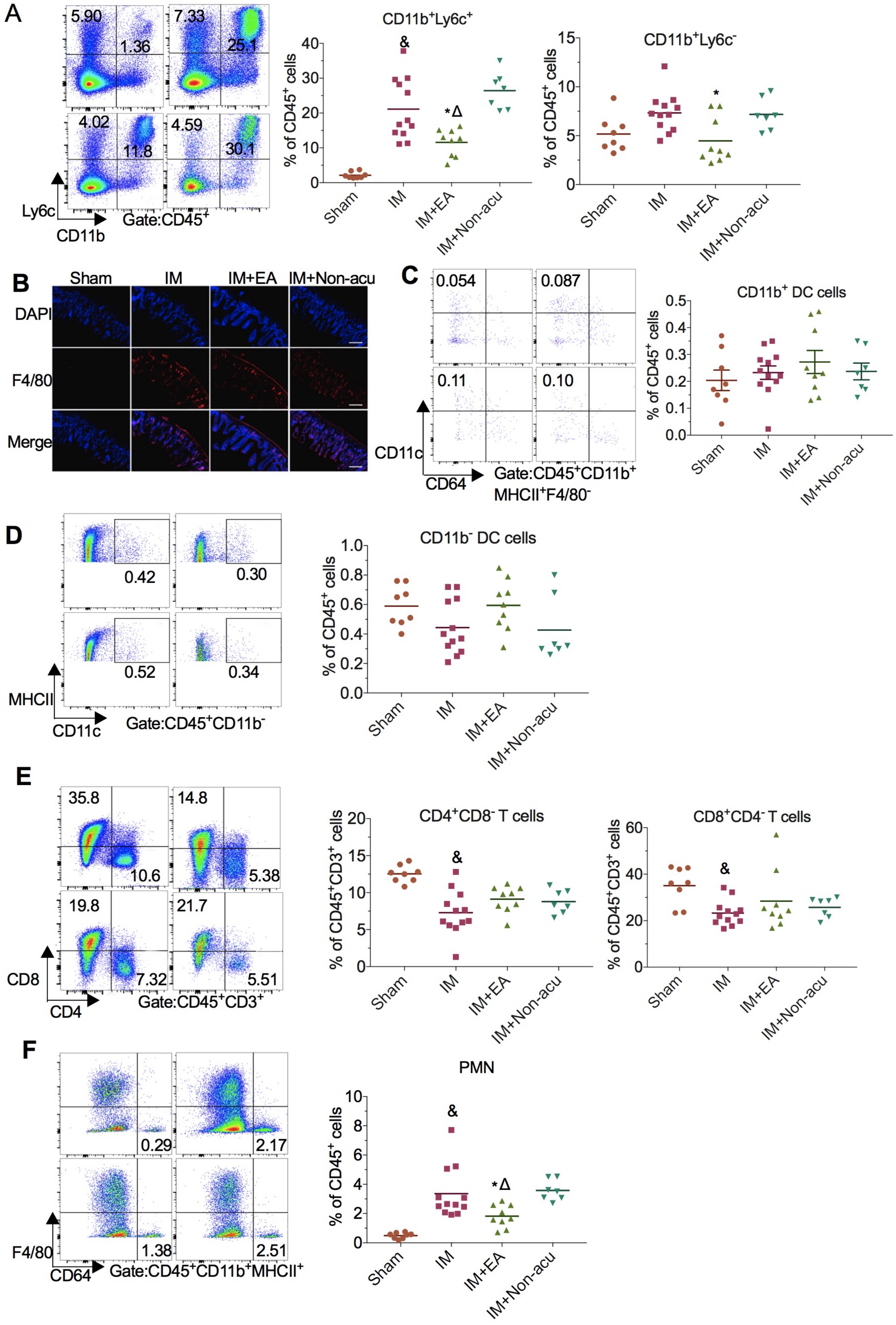

Figure S1 The frequencies of myeloid cells and T cells in four groups by flow cytometry. The frequencies of $CD45^+CD11b^+$ myeloid cells including $CD45^+CD11b^+Ly6c^+$ circulating

inflammatory myeloid cells (A), CD11b+ DCs (C), CD11b- DCs (D), T cells (E) and CD45+CD11b+MHCII-Ly6G+ PMN cells (F) among CD45+ cells by flow cytometry. The active level of macrophages (B) was evaluated by immunofluorescence staining. × 100 magnification. $^{\&}p < 0.05$ versus Sham group, $^{*}p < 0.05$ versus IM group, $^{\Delta}p < 0.05$ versus IM + Non-acu group. DC, dendritic cells; PMN, polymorphonuclear.

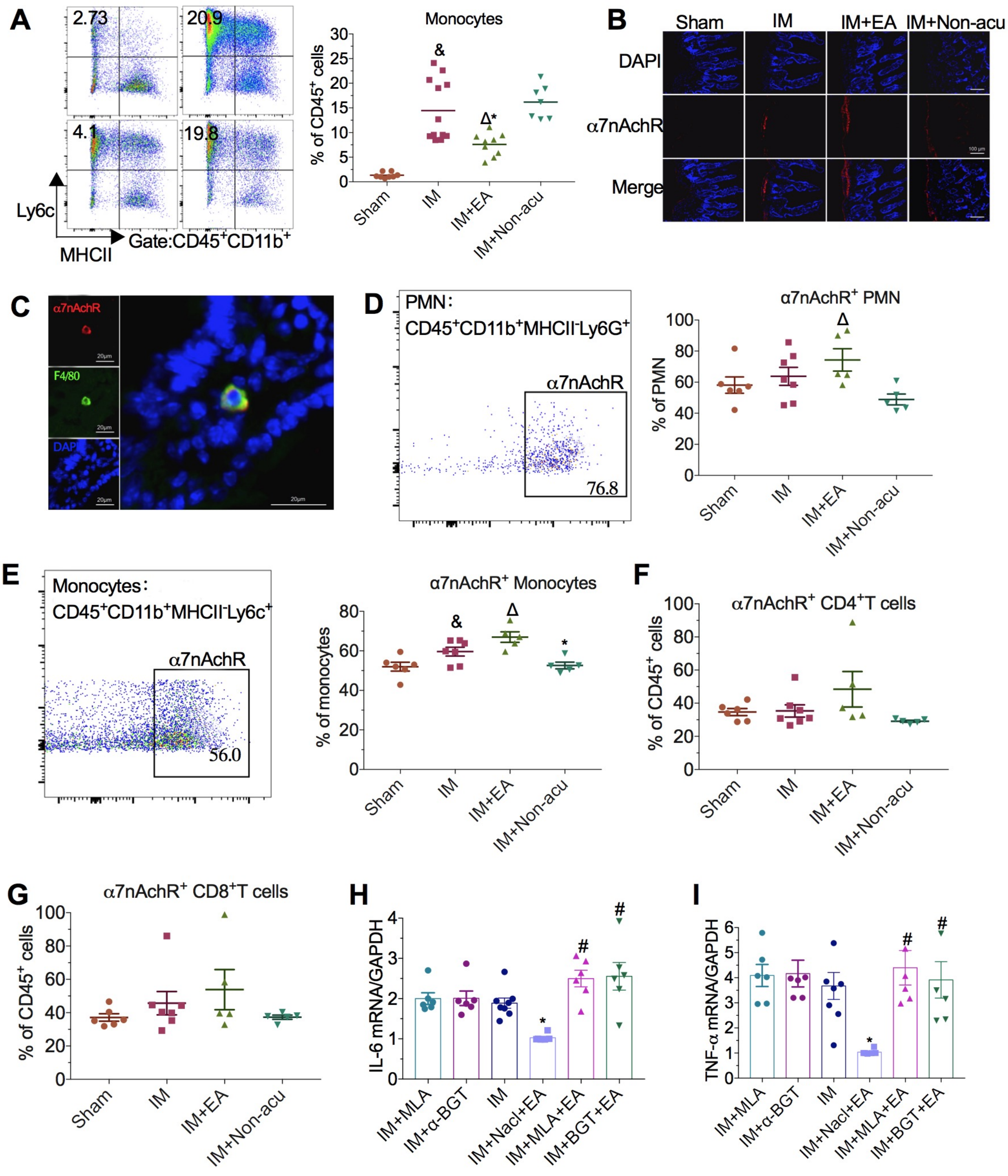

Figure S2 The expression of α7nAChR in PMN and monocytes. The frequencies of $CD45^{+}CD11b^{+}MHCII^{-}Ly6C^{+}$ monocytes (A) among $CD45^{+}$ cells by flow cytometry. The active level of α7nAChR (B) and co-location (C) was evaluated by immunofluorescence staining. × 100 magnification. The frequencies of $\alpha7nAChR^{+}$ in PMN (D), monocytes (E) or T cells (F and G). The expression of IL-6 (H) and TNF-α (I) was analyzed by qPCR. $^{*}p < 0.05$ versus IM group, $^{\#}p <$ 0.05 versus IM + Nacl + EA group. PMN, polymorphonuclear.

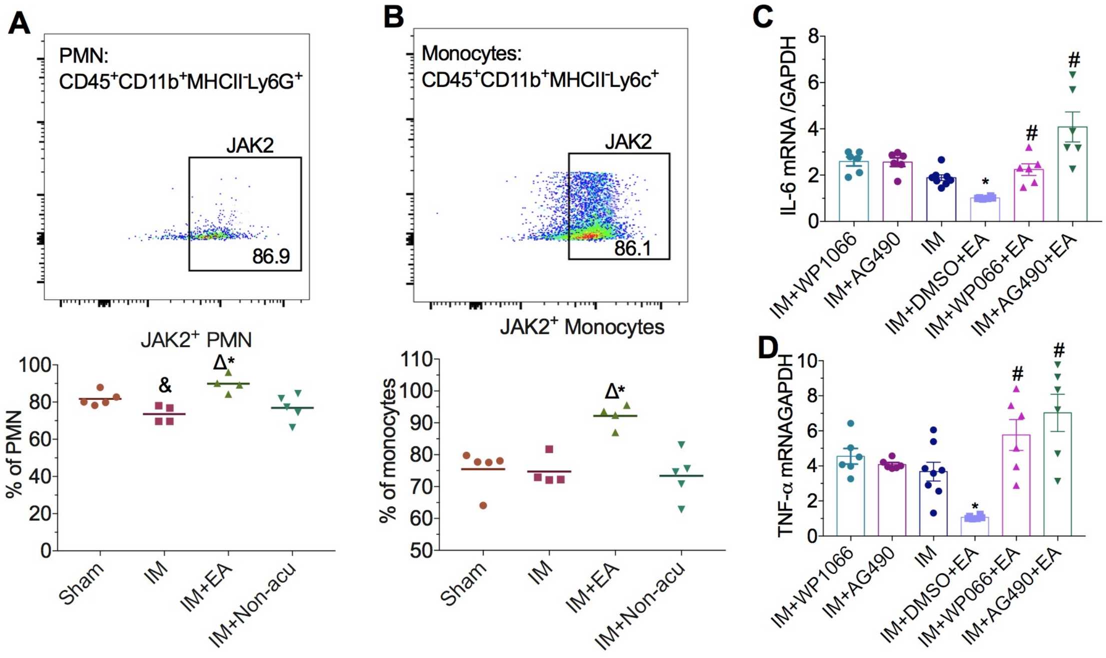

Figure S3 The expression of JAK2 in PMN and monocytes. The frequencies of $JAK2^{+}$ in PMN (A) and monocytes (B). The expression of IL-6 (C) and TNF-α (D) in intestinal muscularis was analyzed by qPCR. $^{\&}p < 0.05$ versus Sham group, $^{*}p < 0.05$ versus IM group, $^{\Delta}p < 0.05$ versus IM + Non-acu group, $^{\#}p < 0.05$ versus IM + DMSO + EA group. PMN, polymorphonuclear.

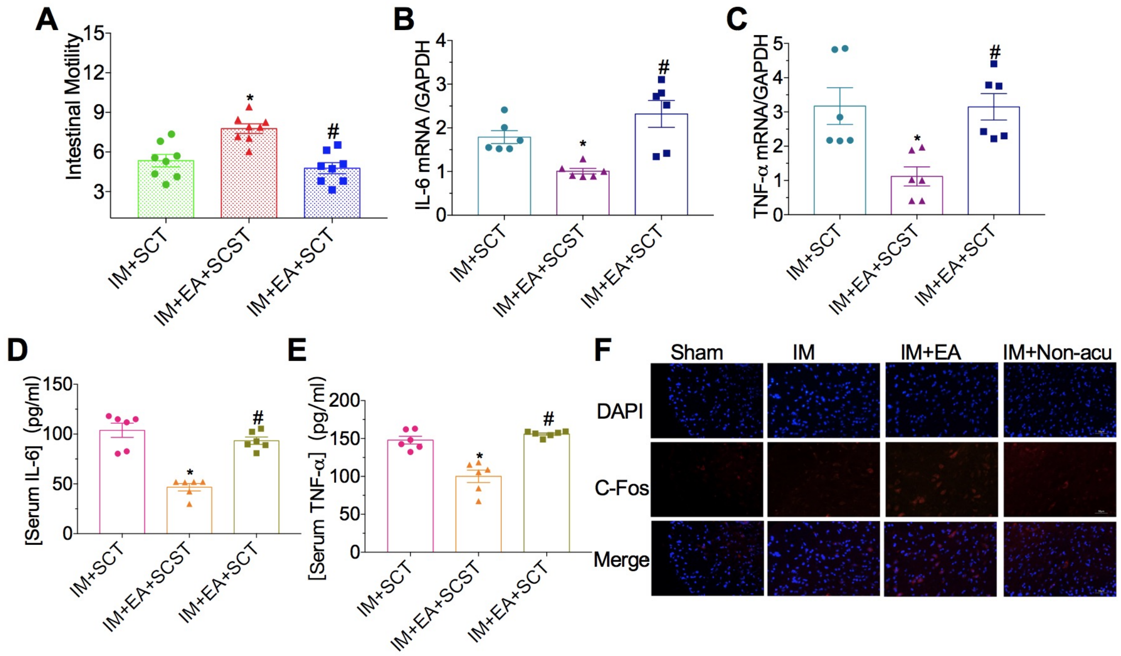

Figure S4 Sciatic nerve takes part in the protective function of EA in POI. (A) The gastrointestinal transit was measured in each SCT groups. The expression of IL-6 (B) and TNF-α (C) in intestinal muscularis was analyzed by qPCR. The expression of serum IL-6 (D) and TNF-α (E) was analyzed by ELISA. (F) The level of C-Fos was evaluated by immunofluorescence staining in spinal cord. × 100 magnification. $^{*}p < 0.05$ versus IM + SCT group, $^{\#}p < 0.05$ versus IM + EA + SCST group. SCT, sciatic neurectomy; SCST, sciatic sham neurectomy.
